# Orbital aspergillus infection mimicking a tumour: a case report

**DOI:** 10.4076/1757-1626-2-7860

**Published:** 2009-09-15

**Authors:** Muhammad Ahsan Zafar, Syeda Sidra Waheed, S Ather Enam

**Affiliations:** 1 Department of Neurosurgery, The Aga Khan University HospitalStadium road, 3500, Karachi, 74800Pakistan; 2 Department of Surgery & Biological Sciences, The Aga Khan University HospitalStadium road, 3500, Karachi, 74800Pakistan

## Abstract

A 14-year-old male presented to the neurosurgical clinic with swelling just above the right eye which had been growing slowly for the last eight years. The swelling first appeared following a non-penetrating trauma eight years ago. On examination it was a non-tender, non-erythematous, firm, round swelling causing marked proptosis and diplopia on downward gaze only. The visual acuity was intact. MRI showed an intraorbital, extraconal mass isointense on T1 and hypointense on T2 imaging. A diagnosis of orbital tumor was made. A white, friable mass consistent with meningioma was resected. However histopathology report later showed it to be an Aspergilloma. The patient was successfully treated with anti-fungal medicine and was disease-free at one year follow-up.

## Introduction

Orbital masses can be neoplastic, infectious or inflammatory in origin. Owing to the confined nature of the orbital space they present with overlapping clinical manifestations. Yet based on some disease-specific symptoms, signs and radiological imaging a definite diagnosis can usually be reached. An indolent growth of a solitary, discrete mass is usually suggestive of a tumor. Fungal infections of the orbit are almost exclusively seen in immune compromised states. In this report, we present a case of an orbital mass of infectious origin with fungal etiology that grew slowly over a period of eight years and had clinical features suggestive of a neoplasm.

## Case presentation

A 14-year-old South East Indian male presented to our tertiary care hospital with a painless, non-tender swelling just above the right eye. The patient, a school-going child from a rural coastal village in Pakistan, was accompanied by his father.

The swelling had developed over the last eight years following a superficial orbital trauma by a cricket bat with no bone or eye involvement. There was marked proptosis of the right eye but no complaints of visual impairment, pain or discharge. Binocular diplopia was present on downward gaze. He had undergone resection of the mass through the superior palpebrae in a rural out-patient clinic previously. At that time, the mass was apparently partially resected. The tissue was not sent for histopathological evaluation and no medical record was available regarding that procedure. Review of systems was unremarkable. The patient had no constitutional complaints, and reported no change in appetite or weight.

Past medical history was remarkable only for the out-patient resection procedure three years ago. No records were available from that time. The patient was delivered at home, at term, after an unremarkable pregnancy, and achieved all mile-stones at appropriate ages. No record of child-hood immunizations was present. There was no evidence of a disease causing immuno-suppression in the patient, and nutritional status was within normal limits. Family history was unremarkable, and he was taking no medications at that time. There was no history of alcohol, tobacco or illicit drug use and the patient was not sexually active.

The patient was at the 50^th^ percentile for height, and 40^th^ for weight, and was in no obvious distress. General physical exam was unremarkable with no lymphadenopathy noted. The neurological exam, including that of the cranial nerves II, III, IV and VI was unremarkable. A non-tender, non-erythematous, moderately hard intraorbital mass was palpable superior-medial to the right eye-ball. The rest of the systemic exams, including head and neck exam with sinuses, were unremarkable.

**Figure 1.**
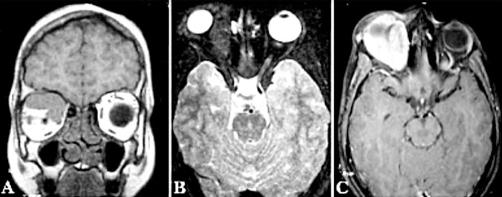
MRI **(A)** T1 Coronal image; an isointense mass located superio-medially in the right orbit, pushing the orbital contents. **(B)** T2 axial fat suppressed image; a hypointense, extraconal mass located medially in the right orbit, causing proptosis. No infiltration in the surrounding structures can be seen **(C)** Post-gadolinium axial image; intense contrast enhancement of the mass.

The MR imaging demonstrated an extraconal mass in the right orbit. The mass was isointense on T1 weighted imaging and hypointense on T2 weighted imaging. Significant mass effect was noted with the orbital contents pushed inferio-laterally. Infiltration in the surrounding tissue or extra-orbital extension was not observed. Post gadolinium T1 weighted imaging showed intense homogenous contrast enhancement (Figure 1).

**Figure 2.**
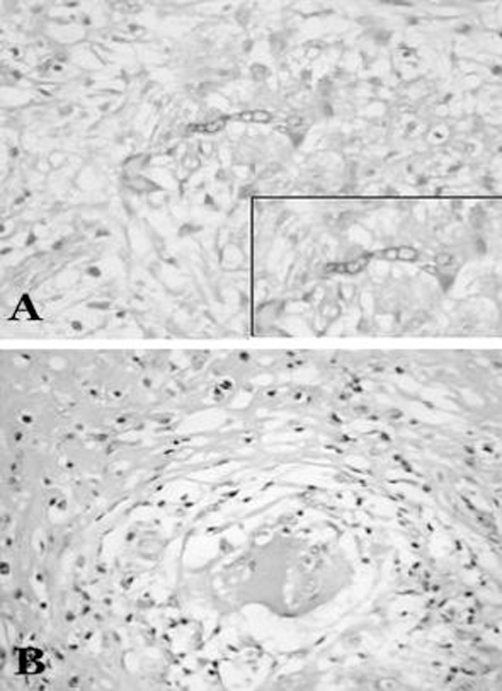
Histology **(A)** Periodic acid schiff (PAS) staining; purplish-red fungal hyphae are seen, surrounded by moderate infiltrate of polymorph leuckocytes. Inset-magnified view of the fungal hyphae. **(B)** H & E staining; large Giant-cell surrounded by leuckocytic infiltrate.

Based on the history, clinical presentation and radiological imaging, a diagnosis of orbital tumor was made. Surgical excision was carried out using the anterior fossa approach. Intraoperatively, the mass was located under the periorbital fascia. After opening this fascia, the frontal nerve and then its branch the supraorbital nerve was identified leading into the mass but then it could not be followed any further. All the muscles including the superior oblique and levator palpebrae superioris were pushed down by the mass. The mass was pearly white, friable and of cartilaginous consistency with no infiltration into the surrounding tissue, resembling a meningioma or neurofibroma. The mass was discrete and seemed to be arising from the supraorbital nerve. The supraorbital nerve is a terminal branch of the frontal nerve which arises from the ophthalmic division of the trigeminal nerve, the fifth cranial nerve. The tissue was sent for histopathological diagnosis which showed chronic granulomatous inflammation with multi-nucleated giant cells and areas of necrosis. Special stains; PAS (periodic acid Schiff) and tissue culture revealed numerous septate fungal hyphae consistent with Aspergillus Fumigatus (Figure 2). There was no evidence of neoplasm on histological studies. The patient was treated with oral itraconazole, 200 mg twice daily for five days and then once daily for 2 months.

At one year post-surgical follow-up there were no complaints of visual or neurological functions. The examination and work-up was unremarkable.

## Discussion

This is a case of a 14-year-old male with an orbital mass, histologically proven to be a Aspergillus infection but clinically suggestive of a tumor.

Primary orbital tumors of the pediatric age group are dermoid and epidermoid cyst, capillary hemangioma and rhabdomyosarcoma [1]; in the adult age group common primary tumors are lymphoid tumor, cavernous hemangioma, meningioma, neurofibroma and schwannoma. Orbital tumors have protean manifestations. The signs and symptoms are related to mass effect of the tumor within the compact orbit. Proptosis and exophthalmos are the most common presentations. Others are restricted ocular movements, lagopthalmos, exposure keratitis, chemosis, visual impairment and diplopia. Most tumors are slow growing therefore the symptoms develop over a period of time. In the case presented here, the swelling had developed in an immuno-competent person over a period of eight years with no acute symptoms. The only complaint was slowly progressing downward gaze diplopia. The indolent course suggested neoplastic nature of the mass.

Orbital infections occur due to spread of infection from paranasal sinuses or direct inoculation due to trauma, surgery or skin infections [2]. Orbital cellulitis is most commonly caused by bacterial infection. Fungal and viral etiologies occur less frequently. Mycotic orbital cellulitis is often seen in patients with uncontrolled diabetes mellitus or other immunocompromised states such as AIDS, malignancy or steroids use. They may be invasive or non-invasive. Fungal etiologies include Aspergillus, Mucor and Cryptococcus species [2]. Patients with orbital infections causing mass effect may present with symptoms similar to tumors. In addition infections may have inflammatory signs and signs/symptoms associated with paranasal sinusitis or intracranial extension. Fungal infections of the orbit may have atypical presentation. There is one report each for orbital fungal infection presenting as optic neuritis [3], sub-periosteal abscess [4], optic neuropathy [3] and orbital apex syndrome [5]. On the other hand, orbital tumors have been reported to present as dysthyroid eye disease [2], ophtalmoplegia [2], epiphora [6] and Duane syndrome [7]. In the case presented here, the patient was immuno-competent, there was no evidence of any inflammatory process on clinical evaluation, and there was no involvement of paranasal sinuses or intracranial extension of the disease. Lack of such findings gave a false clinical impression of orbital tumor.

MR imaging is preferably used to assess the orbital disease as it gives a detailed picture of soft tissue structures, especially in case of fungal infections which have the propensity to extend into surrounding structures. The MRI findings in orbital fungal infections are very characteristic. These include a mass lesion producing hypo-to-isointense signals on T1 weighted and extremely hypointense on T2 weighted with bright homogenous enhancement on post-gadolinium T1 weighted images [8]. Optic nerve sheath meningioma appears as a localized or fusiform enlargement of the optic nerve. T1 and T2 weighted images usually show no significant change in intensity of meningioma compared to the normal optic nerve. Gadolinium images show marked or moderate contrast enhancement [3].

In our case, MRI findings were of an extraconal mass, isointense on T1 weighted and hypointense on T2 weighted with intense homogenous contrast enhancement on post-gadolinium T1 weighted. The literature shows that these findings are characteristic of an Aspergillum infection [8]. The long-standing history, the slow indolent growth, absence of inflammatory signs and no infiltration in the surroundings caused it to be misdiagnosed as a tumor and the findings on operative resection of the mass also seemed consistent with meningioma arising from the supraorbital nerve. Hence, fungal infections should always be kept in differentials of such solitary orbital masses with long indolent growth.
